# Cobalamin in inflammation III — glutathionylcobalamin and methylcobalamin/adenosylcobalamin coenzymes: the sword in the stone? How cobalamin may directly regulate the nitric oxide synthases

**DOI:** 10.1080/13590840701791863

**Published:** 2008-01-10

**Authors:** Carmen Wheatley

**Affiliations:** Orthomolecular Oncology, 4 Richmond Road, Oxford OX1 2JJ, UK

**Keywords:** Glutathionylcobalamin, methylcobalamin, adenosylcobalamin, methionine synthase, methylmalonyl CoA mutase, inducible/neuronal/endothelial nitric oxide synthases, phorphyrin/corrin NOS monoxygenation, cP450, arginine, N^G^-hydroxy-l-arginine, imidazoles, dimethylbenzimidazole

## Abstract

Several mysteries surround the structure and function of the nitric oxide synthases (NOS). The NOS oxygenase domain structure is unusually open with a large area of solvent that could accommodate an unidentified ligand. The exact mechanism of the two-step five-electron monoxygenation of arginine to N^G^-hydroxy-L-arginine, thence to citrulline and nitric oxide (NO), is not clear, particularly as arginine/N^G^-hydroxy-L-arginine is bound at a great distance to the supposed catalytic heme Fe [III], as the anti-stereoisomer. The Return of the Scarlet Pimpernel Paper proposed that cobalamin is a primary indirect regulator of the NOS. An additional direct regulatory effect of the ‘base-off’ dimethylbenzimidazole of glutathionylcobalamin (GSCbl), which may act as a sixth ligand to the heme iron, promote Co-oriented, BH_4_/BH_3_ radical catalysed oxidation of L-arginine to NO, and possibly regulate the rate of inducible NOS/NO production by the NOS dimers, is further advanced. The absence of homology between the NOS and methionine synthase/methylmalonyl CoA mutase may enable GSCbl to regulate both sets of enzymes simultaneously by completely separate mechanisms. Thus, cobalamin may exert central control over both pro-and anti-inflammatory systems.

## Introduction

Over the past 50 years there has been a growing awareness among clinicians and researchers that cobalamin (Cbl), vitamin B12, in all its forms, has powerful effects in inflammation, for a diverse range of pathologies, chronic and acute. Yet, in spite of some notable research demonstrating, for example, that Cbl directly controls the key inflammatory cytokines tumour necrosis factor alpha (TNFα) [1] and interleukin 6 [2], the growth factors epidermal growth factor (EGF) [1] and nerve growth factor (NGF) [3] and, moreover, that Cbl modulates immunity through its effects on CD8+ T lymphocytes and natural killer cell activity [4,5], the exact mechanism of these actions remained a mystery. In two previous hypothesis papers, A Scarlet Pimpernel for the Resolution of Inflammation? [6] and The Return of the Scarlet Pimpernel [7], the latter partly based on novel, supportive research findings [8], I proposed a possible answer. Cbl regulates inflammation by regulating nitric oxide (NO), not, as had previously been thought, by simply acting as an NO ‘mop’, or antagonist, but by regulating NO production and its safe deployment [6–8], through the regulation of all three nitric oxide synthases (NOS), constitutive endothelial and neuronal NOS, (eNOS, nNOS) and inducible NOS (iNOS), while simultaneously, in a mutually responsive complementary manner, regulating key antioxidant systems. Cbl does this indirectly, by promoting the synthesis of NOS substrates and cofactors, heme, arginine, tetrahydrobiopterin (BH_4_), the nucleotides FAD/FMN and NADPH [7] and also glutathione (GSH) status, which is ultimately dependent on Cbl status [7,9,10]. A deficiency of any substrates or cofactors results in ‘uncoupled’ NOS reactions, decreased NO production, and increased or excessive O_2_^−^, H_2_O_2_, ONOO^−^ and other reactive oxygen species/reactive nitrogen species (ROS/RNIS), leading to pathologies of unresolvable inflammation. Cblpromoted GSH in turn favours the formation of more benign NO species, s-nitrosothiols, and reverses, or modulates, the effects of nitrosylation in cell signal transduction.

The marriage of GSH and Cbl results in the formation of glutathionylcobalamin (GSCbl), formed immediately on cell entry from H_2_OCbl^+^ and reduced GSH [11]. GSCbl is a particularly stable intermediate postulated as participating in the formation of the B12 coenzymes methylcobalamin (MeCbl) and adenosylcobalamin (AdoCbl) [12]. No intracellular role has otherwise been established for GSCbl. However, its remarkable stability and increased, but controlled, rate of formation in inflammation [11] suggest that GSCbl may have other intracellular roles and targets than the two Cbl coenzymes methionine synthase (MS) and methylmalonyl CoA mutase (MCoAM). New evidence suggests that GSCbl in particular can selectively promote iNOS and support eNOS NO formation in the early stages of inflammation [8]. Such GSCbl promotion of iNOS and eNOS NO production in the pro-inflammatory phase is seen as a positive event, increasing the efficacy of the immune response [7] while mitigating damage to the host by lowering TNFα [7,8], for example, and eventually signalling resolution and consequent selective inhibition of iNOS and Nuclear Factor Kappa B (NFκB) [7]. Old, overlooked evidence also suggests that in extreme nitrosative or oxidative stress, GSCbl can regenerate activity of enzymes important for eventual resolution, such as glucose 6 phosphate dehydrogenase, which ensures NADPH supply, and of lactate dehydrogenase, aconitase and cytochrome *c* oxidase [7,13].

It seems probable then that GSCbl is more than a go-between that amplifies the formation of MeCbl and AdoCbl in MS and MCoAM catalysis [14]. Perhaps GSCbl may have a role as a ‘border guard’, packaging Cbl in a way analogous to the safe packaging of NO by GSH or other thiols such as S-nitroso-glutathione (GSNO) [15–18] or GSCbl may prevent intracellular NO–H_2_OCbl direct interactions that could have undesirable consequences [7]. Pointedly perhaps, the binding affinity/formation constant of GSCbl (5 × 10^9^ M^−1^) [11] is above that of the supposed NOCbl (1.0 ± 0.5 × 10^8^ M^−1^) [19] formed by proposed NO/H_2_OCbl(II)^+^ interaction. Furthermore, intracellular H_2_OCbl would have the potential to inactivate the two Cbl coenzymes [20,21]. It is true that in ex vivo chemical studies, GSCbl has been shown to interact with NO, apparently yielding Cbl (III)-NO^−^ +GS͘ (gluthationyl radical), although the latter product was not actually verified [19]. However, this reaction was promoted by a ratio of NO 10–20 times higher than GSCbl, which may not be representative of in vivo discrete, intracellular compartment, unbound NO to GSCbl ratios. Moreover, this, and similar studies that subscribe to the Cbl as an NO ‘mop’ paradigm, used to ‘explain’ vasoconstrictor effects of Cbl, may not quite fit the more complex biochemical relationships between Cbl, GSH and NO. GSH, for example, is present in cells at an order of magnitude six times greater than Cbl, a ratio that appears very tightly controlled, and may discourage competition for NO by Cbl. The vasoconstrictive effects of Cbl may equally well be the indirect result of its fundamental promotion of GSH [7], which in turn has a very strong affinity for NO to which it binds, continually reversing the effects of nitrosylation in diverse systems and cell signal transduction. This would include the regulation of vasodilation.

Of course, this postulate may be wrong, or the interaction of GSCbl with NO may, if it occurs endogenously, have some other as yet undefined role. It has also been suggested that GSCbl may act as a reservoir for intracellular Cbl III [22]. This is a possibility. However, GSCbl may also exist as a modest reservoir for GSH to recombine with NO, as needed, thereby modifying NO's effects towards positive outcomes, if the balance between snitrosothiols and RNIS shifts too far towards the latter. Perhaps this even occurs in the very process of NO formation? Hence, perhaps the paradox of GSCbl's increasing formation rate constant with decreasing pH, alongside an increasing equilibrium constant with increasing pH? [11]. Perhaps too GSCbl may provide this GSH for NO modulation in protein regions not accessible to unbound GSH itself?

The mention of GSCbl and protein accessibility brings us to an interesting unanswered question: whether GSCbl forms any kind of protein link outside the catalytic interactions with the Cbl coenzymes MS and MCoAM? This hypothesis will sketch the brief outline of a wildly speculative idea, which may prove to have a chemical basis. The scheme presented in The Return of the Scarlet Pimpernel [7] suggests how Cbl, via its two coenzymes, is responsible for the supply of all the substrates and cofactors of the NOS, and in the process also keeps the redox balance and promotes the more benign species and effects of NO, and thereby indirectly regulates the NOS. A recurrent theme of this new paradigm shows Cbl acting as a ‘back-up disc’ for biological systems. Is it possible that Cbl may have a potential direct regulatory interaction with the NOS and that such a direct as well as indirect, regulatory interaction might serve to enhance or, once more, back-up or modulate the effects of the various primary promoters and inhibitors of the three NOS? Is it possible perhaps even that the corrin macrocycle can stand in for the porphyrin in the NOS heme protein, which might serve principally as a template, or back-up for the corrin? And that it is the Co that is involved in the oxidation of arginine and N^G^-hydroxy-L-arginine (NHA)? The relatively open ‘baseball mitt’ structure of the NOS oxygenase (NOSox) domain and the funnel-shaped active channel might be designed to fit in both the two porphyrins and the two catalytic corrins. The increased arrival of Cbl intracellularly in inflammation [6] and the increased rate constant but controlled formation of GSCbl [7,11] may be partly to this end. Is it also just coincidental that Transcobalamin II receptors (TCIIr) expression is increased by interferon β [7], and that without IFNα/β iNOS NO production declines considerably? [23] One, perhaps rather fanciful, possibility may be a direct link of GSCbl with the dimethylbenzimidazole (DMBI) ‘base-off’ to the Co and ‘base on’, via the N^3^ of its imidazole, to the heme, as a sixth iron ligand in the NOSox domain.

## NOS structures

**Figure 1.**
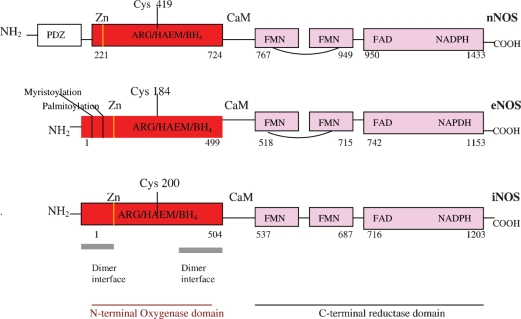
Human neuronal (nNOS), endothelial (eNOS) and inducible nitric oxide synthase (iNOS) domain structure (PDZ domain, named after homologous domains in three proteins: PSD-95, DH/g, ZO-1).

**Figure 2.**
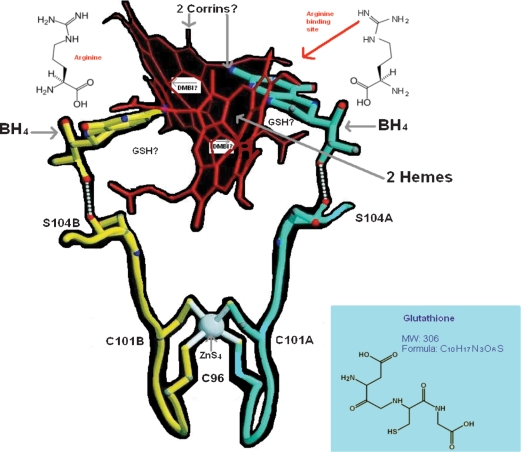
Model of nitric oxide synthase oxygenase (NOSox) dimer with approximate scheme for hypothetical glutathionylcobalamin (GSCbl) links in relation to the two hemes, ZnS_4_, BH_4_, arginine. Ser 104 is in the loop with the Cys ligands and H bonds to the C6 side chain of BH_4_.

The structures of the three NOSox domains, solved by X-ray crystallography and limited proteolysis [24–29] are so similar to each other that drug designers who subscribe to the NO overproduction in pathology paradigm find it hard to see how the NOS mightbe selectively inhibited. It may be likely, however, that this homology is not accidental, from the point of view of endogenous regulation of the NOS. Rather, this homology might suggest that the endogenous regulator could be like a single key that fits similar but slightly different locks, the key being the DMBI of GSCbl, and the locks the NOSox domains. But there are, in fact, some slight structural differences between the three individual NOSox and the three individual NOS reductase domains, which may alter the effects of the DMBI key, so that it promotes some NOS and inhibits others. Interestingly, the main difference in the NOSox is not between the inducible and constitutive NOS, but between nNOS, on the one hand, and eNOS and iNOS, on the other. This grouping difference is already apparent in the NOS's molecular weight, with nNOS at 165 kDa and eNOS and iNOS almost identical at 133 and 131 kDa, respectively [30]. (Intriguingly, these weights are close to the two Cbl coenzymes: MS 136 kDa [20] and MCoAM 150 kDa [21].) The NOS gene structure and size also indicates this division: nNOS, 29 exons/28 introns, complex structural organization and locus over a region of > 200 kbp; eNOS, 26 exons/25 introns, 21–22 kbp; iNOS, 26 exons/25 introns, 37 kbp. The overall primary domain structure with amino acid residue sequence positions for the individual substrates and cofactors of the three NOS may be seen in Figure 1. The cysteine residue ligating the heme to the calmodulin (CaM)-binding site is highlighted in all three NOS, and here again there is a difference between nNOS ligation at Cys419 and eNOS/iNOS at relatively close Cyst184 and Cyst200, respectively. The amino acid loop insert in the middle of the eNOS/nNOS FMN binding reductase domain, however, differentiates them from iNOS. This loop is thought to be auto-inhibitory [31] and acts by destabilizing CaM binding at low Ca^2+^ concentrations and thus inhibits electron transfer from FMN to heme, in the absence of Ca^2+^/CaM binding [30]. As discussed previously, individual NOS reactions that catalyse the five electron monoxygenation I and II processes involved in NO production are more or less ‘coupled’ in respect of reduction by the flavins and NADPH [7]. A study of all three isoforms using the artificial electron acceptor, cytochrome *c*, and comparing them with NADPH-cP450 reductase-catalysed cytochrome *c* reduction, showed minimal turnover for eNOS/nNOS in the absence of Ca^+^/CaM. On addition of Ca^+^/CaM nNOS showed a 10-to 15-fold increase in cytochrome *c* reduction, 1.6 times the rate of NADPH-cP450 reductase reduction. Although eNOS cytochrome *c* reductase activity increased by more than twofold, on Ca^2+^/CaM addition, its activity was only about 16% of the NADPH-cP450 reductase. iNOS, in the absence of Ca^2+^/CaM catalysed cytochrome *c* reduction at the same rate as nNOS+Ca^2+^/CaM [32]. This gives a ranking for the individual isoform reduction potentials: iNOS=nNOS≫eNOS [32]. Another study in which electron transfer to the heme oxygenase domain was measured by reduced CO difference spectroscopy, which requires reduced heme, found that eNOS was the most ‘tightly coupled’ isoform, with NADPH reducing the heme as much as dithionite, much superior to the 70% reduction of heme by nNOS and 30% by iNOS [32]. Other non-structural differences between the constitutive and inducible NOS may also have a bearing on potential regulation by GSCbl and its DMBI. Dimer assembly in iNOS appears to involve only the oxygenase domain, whereas in eNOS/nNOS it involves interactions, within the reductase domain, and between the reductase and oxygenase domains across the dimer [30]. iNOS is consequently more dependent on BH_4_ binding for its dimeric assembly than eNOS/nNOS [33]. BH_4_ in the iNOSox folds the central interface region in a novel αβ fold, to create a 30Å-deep, funnel-shaped active-site channel and tilt the heme so it is available for interactions with the reductase domain [30] (Figure 2). All three NOS, of course, also require insertion of heme [34] and binding of the L-arginine substrate, as well as the cofactor, BH_4_, for dimerization and activity, and so, setting aside the structure of NOS isoforms and their internal oxidation reduction kinetics, individual NOS binding affinity for L-arginine might also be relevant to how GSCbl and its DMBI may intervene, as may slight variations in the accessibility of the heme, which is buried deep in the protein's interior in the distal pocket, making extensive van der Waals interactions with hydrophobic and aliphatic side chains. So there is little solvent accessibility to the heme, except for one of its propionates, and a large, 750Å^3^ [33] substrate/cofactor access channel, which allows solvent access to both the active site, heme and BH_4_.

## Imidazoles and NOS promotion/inhibition

The hypothesis that the GSCbl DMBI may have the potential to directly regulate the NOS is drawn from studies of compounds that promote or inhibit NOS, specifically imidazoles and N^1^-substituted imidazole derivatives or analogues, which, like CO, NO, CN, have the capacity to bind directly to the pentacoordinate heme iron as a sixth ligand, preventing O_2_ binding [24,35–39]. Arginine, a bulkier molecule with non-bonded electrons, does not directly bind to the heme iron, but binds to the protein in a network of hydrogens, near the distal heme pocket, and lies with its central N-guanidine, 3.8 Å away from, and coplanar to, the heme [30,40]. X-ray crystal data show that this dense network of hydrogen bonds orients L-arginine and NHA rigidly in relation to the heme, so that NHA is bound as the anti-stereoisomer, with its hydroxylimine oxygen and guanidinium carbon distant from the iron (4.3 and 4.4 Å, respectively) [40]. Moreover, the porphyrin ring is in a non-planar concave configuration, with the bowl facing into the NOS distal pocket, where the heme is buried. This configuration is not affected by BH_4_ and in eNOS is similar to that of the heme in peroxidases [33], which utilize a histidine residue as the proximal ligand. The iNOS crystal structure reveals that the heme plane facing the proximal thiolate ligand is flipped 180°, exactly opposite to that in cP450s with which otherwise NOS share certain similarities, such as NADPH, FAD/FMN and monoxygenation [24]. The positioning of NHA so far from the heme iron poses a puzzle in respect of its aerobic oxidation to citrulline and NO, because this oxidation reaction requires three electrons but consumes only one NADPH reducing equivalent and apparently, although not conclusively, one from NHA. It is also not clear whether the NOS FeIII heme is reduced by NHA or the NADPH-derived reducing equivalent to initiate the second step [40]. The bulk of the evidence suggests that NHA cannot reduce FeIII heme [40]. X-ray crystal data also preclude direct ligation of NHA to FeIII heme [40]. The source of the missing electrons is not clear, and both a nucleophilic hydroperoxo-Fe(III) heme, the oxenoid species, (oxo-Fe(IV)(PN^0+^) of P450 oxygenase reactions, or a radical-type auto-oxidation mechanism have been proposed as the oxidizing intermediates, but none has yet been definitively confirmed [40].

The crystal structure of an iNOSox monomer with heme inserted shows that two molecules of imidazole can bind within the heme distal pocket, one ligating the heme iron and the other the carboxylate of murine Glu 371, and Glu 377 of human iNOS, which also binds L-arginine's guanidino nitrogens [24,38]. Imidazole and I-phenylimidazole promote iNOS dimerization at the same rate as L-arginine and BH_4_, suggesting that there are two ways of promoting iNOS dimerization [35]; firstly by a direct FeIII^+^ interaction of imidazoles, or relatively simple, non-bulky imidazole, such as I-phenylimidazole derivatives, which have an accessible N^3^, that ligate the heme iron, followed by L-arginine, BH_4_ promoter binding. Alternatively, by initial substrate and promoter, L-arginine, BH_4_ binding, followed by productive subunit dimeric interaction involving imidazoles [35]. These imidazoles are small enough and hydrophobic enough to fit into the heme distal pocket, and are strong true heme ligands, which produce a type II low-spin heme spectrum with *K*_d_ in the millimolar range [24,39]. (L-arginine and BH_4_ after bonding to the heme protein can effect a gradual shift to the five co-ordinate high-spin heme.) Dimethylimidazole also has such an effect [24,37–39]. The question is: can GSCbl's DMBI do this to some degree? (The bulky benzene ring may modulate matters.) If it can also act as a sixth FeIII^+^ ligand, the DMBI may in effect prevent the FeIII^+^heme from being reduced, thus presenting the possibility that the Co in GSCbl can be reduced in its place.

The discovery that there are two ways of promoting iNOS dimer assembly at the same rate might tie in with the idea that GSCbl and its DMBI may play a regulatory back-up role in the NOS dimer assembly, or, indeed, a central one in catalysis. In the former scenario, for instance, if arginine is relatively low, due to diet or other factors, a direct effect of GSCbl on iNOS dimer assembly, with some degree of interplay between the above-mentioned two modes of dimer assembly, might maximize the impact of well-coupled, relatively low L-arginine. If one subscribes to The Return of the Scarlet Pimpernel hypothesis' view of the importance of good iNOS function and higher NO production in inflammation, this backup role of the DMBI could be critical in acute immune defence scenarios, preventing pathologies of unresolvable inflammation, and also in non-acute scenarios such as in the maintenance of the protective functions of continuously active iNOS in lung and retina [7]. Such a mechanism would also have implications for the sustained promotion of eNOS in inflammation, where there may be increased competition between eNOS and iNOS for L-arginine. In other words, it would enable GSCbl's DMBI to simultaneously promote both iNOS and eNOS. nNOS is in a class by itself because it appears that imidazoles paradoxically inhibit nNOS [39], just as they do cP450 and catalase [41]. This inhibition of nNOS is non-competitive with arginine and BH_4_, and negatively impacts on Ca^2+^/CaM-dependent consumption of NADPH [39]. By contrast, in eNOS, L-arginine binds in a manner competitive to imidazoles, including 2-methylimidazole and 4-methylimidazole [37]. Such variations in binding affinity of the two Ca^2+^/CaM binding dependent NOS for the arginine substrate and imidazoles, or the varied degrees to which these enzymes are coupled, together with slight structural variations in their oxygenase domain, the different cysteine residues (cys419, cys184) that ligate the heme to the Ca^2+^/CaM binding site in nNOS/eNOS for example, or known slight variations in each of the auto-inhibitory loops of eNOS/nNOS flavins in the reductase domains, or of the C-terminal tails of all three NOS, which differently regulate electron flow [31,40,42,43] or, again, variable N-terminal domain swapping affecting the size of dimer interfaces [31,43], might all explain how two such homologous isoforms may be differently regulated by the same agent. Moreover, it might be that iNOS alone may be regulated by Co-oriented oxidation of arginine/NHA, whereas eNOS/nNOS are catalysed as traditionally understood, via FeIII^+^reduction.

## Cbl's DMBI ‘false’ nucleotide tail: more than just an evolutionary relic?

The DMBI of MeCbl and AdoCbl surprised B_12_ chemists' expectations for its proposed role in Cbl coenzyme catalysis, by turning out to be base-off to the Co in the respective MS and MCoAM enzymes [20,21,44]. It had been surmised that the DMBI of MeCbl and AdoCbl would play a role in the coenzyme-bound state where it would directly control the reactivity of their upper axial ligands. Instead, in both cases the Co swaps the DMBI for a link to the N^2^ of the imidazole of both proteins' histidine residues, 759 and A610, respectively, thus allowing the proteins to modulate substrate reactivity [20,21,44]. Meanwhile, the DMBI is extended and bound deep in a narrow hydrophobic pocket between the β-sheet and the C-terminal helix of the respective proteins [20,21]. To B_12_ chemists this appeared almost useless, a mere ‘anchor’. But what if it is, in fact, a firmly sheathed sword? Both MS and MCoAM are activated by reduction of their respective Cbls, whose upper axial ligands form highly reactive carbon–cobalt bonds that are easily cleaved. This lability has a distinct relationship to the length of the lower Co–N axial ligand, with the highly reactive alkyl-Co bonds of Me and Ado having longer lower axial bonds (2.20±0.03→2.21 Å), whereas more stable Cbls with strongly bonded upper axial ligands, such as CN or OH, have somewhat shorter Co–N bonds to the lower axial ligand (2.15±0.03Å–2.14±0.03) [45,46]. The notably stable intermediate, GSCbl, of course, falls into the latter category (2.15±0.03 Å) [46]. If we posit that GSCbl has a back-up, or supplementary, modulatory role, or even a central role, which involves some direct interaction with the NOS isoforms, this interaction will probably differ from that of MeCbl/ AdoCbl with MS/MCoAM, as GSCbl is considerably less reactive.

Before the structures of MeCbl/AdoCbl bound MS and MCoAM were elucidated, B_12_ chemists were expecting to find globin or nucleotide-binding folds in the proteins [20], because of the analogy between the porphyrin of heme and the corrin. In the event, MS and MCoAM showed little homology with proteins that bind heme or nucleotides. Nevertheless, perhaps the true home of Cbl's so-called ‘false’ nucleotide, the DMBI, is actually in the NOS heme proteins, deep in a hydrophobic region of the NOS distal heme pocket, and the difference in homology between the enzymes is important because it enables Cbl to affect and regulate both sets of proteins without mutual interference. It is clear also that different Cbls have different biological effects, for all the many different reasons hitherto discussed [7]. But GSCbl is a go-between. In this hypothesis it may simultaneously modulate both sets of proteins, decreasing or increasing the activity of MS and MCoAM together with the formation of MeCbl and AdoCbl to balance the effects of its early promotion of NO in inflammation, a promotion that may be both indirectly modulated as discussed in The Return of the Scarlet Pimpernel [7], or directly modulated by GSCbl's base-off DMBI possibly extended in a new conformation, in the funnel of iNOSox/eNOSox domains with the N^3^ of the imidazole then ligating the heme (Figure 2). Even the established ‘upward’ deformation of the corrin ring found in short Co–N bonded Cbls [45], such as GSCbl, might facilitate the fit with the funnel shape of the NOSox dimer, where the corrin ring might lodge parallel to the porphyrin of the heme down below. This upward deformation of the corrin macro-cycle may be a mimic of the porphyrin's concave bowl, above which it may lie, linking the FeIII below with the N^3^ of the DMBI, while the Co is almost offered to BH_4_. Given that the NHA, like its precursor arginine, is bound at a great distance to the heme iron, as the anti-stereoisomer, in this scenario it would, in fact, be facing, and closer to the corrin, like BH_4_, and this may be of some significance. The substrate arginine restricts O_2_ binding, and Raman data show that bound 2 points away from the substrate [47]. Thus, the distal oxygen of theoretically FeIII^+^ bound O_2_ would be at a great distance from the NHA hydroxyl group. Moreover, the orientation and distance between Fe(III) heme and guanidinium carbon do not favour a proposed peroxonucleophilic attack [40]. It is once more tempting to speculate whether it is not GSCbl that completes the cryptic oxidation of NHA to citrulline and NO? Maybe GSCbl can partially stand in with NADPH as a reducing equivalent? If this were so, a deficiency in Cbl would definitely result in uncoupled NOS reactions, quite apart from the impact of Cbl degrees of deficiency on supply of substrate and cofactors, the absence of which also uncouples the NOS [7]. Ligation of the heme FeIII by GSCbl's DMBI may prevent heme activation and act as a signal, facilitating the reduction of GSCbl to Cbl II instead, followed by activation of O_2_ in tandem with BH_4_, so that NHA is generated from arginine, and then complete oxidation of NHA to NO and citrulline occurs.

## Catalysis of NOS, cP450, MS and MCoAM

Since the observation of the radical species of BH_4_, BH_3_, in NOS, the hypothesis was hitherto advanced that a non-heme metal ion, possibly a non-heme iron, might be the intermediate that catalyses oxidation of L-arginine to NHA [48] in analogy to BH_4_-dependent amino acid hydroxylases, and that because BH_4_-free NOS makes NO^−^, not NO͘, and does not catalyse any reaction with bound arginine, BH_4_ must participate in some way in l-electron chemistry [48]. (Among the candidate metals, Co^3+^ was found to be inhibitory, but this may not detract from the GSCbl hypothesis as the Co^3+^ was deployed as a Cl^2+^ salt [49], a simple compound that would bear little relation to the structural complexity and consequent effects of Co^3+^ in GSCbl.) This less orthodox view had been challenged, both by the discovery that 5-methyl BH_4_ supports the NOS reaction, but not 2 activation, and by laser atomic emission, metal ion analysis, which shows the only metals present in NOS are calcium, zinc and iron. This analysis, however, was done in isolated eNOS mutants. Similarly, another analysis of the NOSox crystal structure that showed no non-heme iron, or other transition metals, bound was carried out with a His-tag fusion protein purified by an Ni resin, and consequently exposed to a high concentration of imidazoles, which would prevent any such binding [25]. The structure of the NOSox active channel shows a large area (750 Å^3^) of solvent, which it has been surmised may accommodate an as yet unidentified ligand. This leaves room for the GSCbl hypothesis. Moreover, even with respect to monoxygenation I, the hydroxylation of L-arginine to NHA, the evidence for the participation of the heme iron is indirect. It is an assumption made largely from its presence in the enzymes, essential for dimerization, and by analogy to the heme role in cP450 catalytic reactions, an analogy further prompted by the sequence homology of NOS and cP450 reductase domains, and shared FAD/FMN content. Yet, if one posits GSCbl as the catalyst in NOS monoxygenations I and II, it may be that the two heme irons are in fact principally anchors for GSCbl, and that a non-heme iron is involved in monoxygenation I, and the Co^3+^ of GSCbl is involved in monoxygenation II. Alternatively, it is also conceivable that the heme iron is involved in monoxygenation I and is cyclically deactivated by GSCbl's DMBI so that the Co^3+^ can complete monoxygenation II, then, as the NO displaces the DMBI from the heme iron, the process begins again.

There is also a possibly fruitful, Cbl enzyme analogy to be made with respect to NOS BH_4_ and GSCbl. In MS catalysis, MeCbl is bound to residues His 757, Asp 75, which are protonated. Deprotonation promotes demethylation and the formation of the radical, Cbl I, and deprotonation of His 757 also increases the nucleophilicity of Cbl I to facilitate its attack on N^5^-methyltetrahydrofolate [20] and consequent methylation of Cbl I to MeCbl III, a cyclical process. N^5^-methyltetrahydrofolate (NMTHF) is a pterin family member, like BH_4_. Maybe GSCbl III in the NOS is able to harvest an electron from BH_4_ then return it following NHA hydroxylation? If so, this may be the reason for the observed BH_4_–BH_3_ recycling, which has always mystified. In yet another analogy, His and Asp residues 610 and 608, respectively, are also important to MCoAM AdoCbl binding and catalysis [21], and therefore it may be worth investigating if the equally close spatially related His 652, Asp 650 in the vicinity of BH_4_ in NOS may be similarly a potential binding site for GSCbl with equal, hypothetical, catalytic potential.

The benzene ring and dimethyl residues of the DMBI may, as noted earlier, have some steric modulatory effects in the active site channel that result in the DMBI being less strongly inhibitory — for nNOS — and/or less strongly promotional — for iNOS, eNOS — than simple imidazoles. In this general scheme, the GSH of GSCbl may also be proximal to the surprising zinc tetrathiolate bridge at the bottom of the dimer interface, 21.6 Å from each heme, and 12 Å from each BH_4_ site, that plays a structural role in NOS quaternary architectural assembly, protects the BH_4_ binding site and possibly provides a docking site for the reductase domain, as the Zn is a strongly positive electrophile [33,50]. This role may be deregulated by known NO ejection of zinc and/or modulated by the formation of disulphide bonds between the symmetry-related cys 115 residues [48–50]. The hypothetical proximity of the GSH in NOSox bound GSCbl (Figure 2) could enable it to keep the zinc bound for activity, and prevent deactivation, by inhibiting disulphide bridge formation, a function that could equally well be accomplished by free GSH perhaps, assuming it had access, which it may not. Of course, the solvent is also a strongly reducing environment, and the GSH of GSCbl may instead have a role in modulating the balance of NO species production in NOS, consistent with its global role of (Cbl-supported) GSH NO packaging discussed previously [7]. Alternatively, the possibility exists that the GSH of GSCbl may form a triad relationship with the zinc and cysteine residues that modulates the rate of NO production and/or release. Nitrosation reactions at thiol residues co-ordinated to metal centres are seen as possible functional switch mechanisms [50]. (This has implications for Cbl–GSH regulation of transcription factors that contain zinc in their DNA-binding domains, for example, the zinc finger of NFκB.) Moreover, cyclic activation/ deactivation of the zinc tetrathiolate by NO/GSH might be synchronous and linked to Cbl III/Cbl II–BH_4_/BH_3_ cycling during NO production. (This would have some analogy to Cbl III–Cbl I recycling during MS catalysis.) The latter possibility may exist because one of the zinc ligands, Cys 101, is only two residues away from Ser 104, which H-bonds directly to the BH_4_ hydroxyl side chain [33] (Figure 2). Moreover, Val 106 of the polypeptide chain forms a direct unbonded contact with BH_4_. Thus, it has been concluded, that any disruption of the zinc or its ligands will result in distortions of this region of the polypeptide chain, diminished affinity for BH_4_ and arginine binding, and loss of protein stability and catalysis [33]. Also of possible relevance to the putative GSCbl/BH_4_ relationship is the demonstration that a thiol, and an inhibitor of the NOS, S-ethylisothiourea, is paradoxically able to promote BH_4_ binding, and, in the absence of BH_4_, binding of the structurally analogous arginine [51]. This finding hints at the possibility that the GSH of GSCbl, although not an inhibitor, may as a thiol have an identical endogenous role, with respect to BH_4_ binding.

As GSCbl elsewhere in the cell would also be simultaneously promoting AdoCbl and MCoAM to counter the potential negative effects of NO overproduction, it would eventually ensure the arrival of increasing amounts of arginine and BH_4_, which could then displace GSCbl's DMBI, in the first ‘back-up’ scenario. Alternatively, in the latter, more central scenario, the DMBI may eventually be displaced by NO when it begins iNOS inhibition, in keeping with the view of GSCbl as the catalyst of NHA oxidation, outlined above. Final NO ejection of the zinc, and reformation of GSCbl (III), may then lead to iNOS dimer degradation. Thus, it may be that the CoIII in the corrin ring can stand in for the FeIII in the porphyrin ring, which it so closely resembles. (Whereas the DMBI may regulate NO and vice versa, as NO is remarkable among small molecules for its ability to displace trans-imidazole ligands.) This scheme is very roughly sketched out, with much fine structural and mechanistic detail unknown and still to be adumbrated, if proven. For perhaps, after all, this latest of the three Cbl hypotheses is just a redundant fantasy. Or perhaps not. Absence of proof is not proof of absence. According to legend, the Scarlet Pimpernel was very elusive indeed.

## Testing the Scarlet Pimpernel hypotheses

A Scarlet Pimpernel for the Resolution of Inflammation? [6] discusses aspects of testing the general hypothesis that Cbl plays a central immunoregulatory role, in the clinic, for systemic inflammatory response syndrome/sepsis/septic or traumatic shock. Since its publication, some pre-clinical studies have already been performed [8] and more are underway. However, as The Return of the Scarlet Pimpernel [7] has reformulated and expanded the original hypothesis, focussing it on Cbl central regulation of the NOS and complementary regulation of key antioxidant systems, it is clear that, if there is truth in this new paradigm, there may be some very widespread and significant implications not only for the treatment of pathologies of unresolvable inflammation such as sepsis, but for other intractable disease, such as malaria, HIV, diabetes, antibiotic-resistant tuberculosis, Alzheimer's disease, methicillin resistant *Staphylococcus aureus* (MRSA), viral epidemics and cancer, where NOS malfunction and iNOS depression may be implicated. In the contrarian view of the ‘Scarlet Pimpernel’, there is no need to design selective inhibitors of the NOS. Indeed, this may be a venture just as ill-fated as the clinical use of unselective NOS inhibitors has proven to be. Instead, by using existing, relatively well-tolerated, immune-priming drugs, principally the interferons α,β,γ, and possibly certain others, such as APO2-L-Trail, in combination with pre-and concurrent treatment with very high — up to 5 g, or over — doses of Cbl, principally perhaps GSCbl, but certainly the already pharmacologically safe and licensed OHCbl, it may be possible to stimulate or reawaken the native endogenous NO regulation mechanism of the immune system for resolving inflammation. In diseases such as malaria and cancer, where parasites and tumours evade immune surveillance and flourish by deregulating the NOS, consequently damping down immunity, high-dose Cbl and interferon α βmight prove curative, by enabling the body to mount a strong, targeted response. As such therapy is, in the coinage of the great Linus Pauling, an ‘orthomolecular’ approach to the treatment of disease, this treatment should be supported with a broad spectrum vitamin, mineral, essential fatty acid supplement, and followed up by maintenance of good nutritional and Cbl status.

However, although there are sufficient human, animal and in vitro pharmacological safety and efficacy data on Cbl to justify clinical studies in sepsis [6], there is a need to do considerable pre-clinical studies for the other aforementioned diseases, and equally to investigate the substance of ‘The Return of the Scarlet Pimpernel’ and ‘The Sword in the Stone?’ thoroughly in the laboratory. Study of the impact of Cbl, particularly GSCbl, in both the pro-and anti-inflammatory phases of immune defence with respect to all three NOS and antioxidant systems has already commenced [8], but there is also a need for detailed molecular investigation with crystallography and/or limited proteolysis of the possible direct impact of GSCbl on all three NOSox domains. A related study might also consider whether GSCbl's DMBI plays a direct regulatory role in nitric oxide dioxygenases (NODS), such as flavohaemoglobin, haemoglobin and myoglobin, which have a high affinity for binding NO, and are, together with Cbl, also crucial to NO homeostasis by continually combining O_2_ with ‘free’ NO to form inactive/non-toxic nitrate (NO_3_) [52]. If direct Cbl regulation of NODS via the DMBI is indeed the case, then one might also surmise that the antibiotic action of NO/GSNO may be further enhanced by the DMBI of AdoCbl on TCIs released in the presence of bacteria, by analogy with the known inhibition by imidazole antibiotics of NODS, such as microbial flavohaemoglobin [53], which contain large hydrophobic heme pockets capable of sequestering bulky aliphatic lipids and imidazole N^1^ substituents [53]. A corollary of the foregoing arguments might also be direct, as well as indirect, Cbl regulation of all heme proteins in a complementary manner: Cbl as the ultimate transcription factor.

It would also be of interest to measure activity in vivo, in various pathologies of unresolved inflammation, with and without Cbl, of key enzymes, such as, MS and MCoAM; aconitase; cytochrome *c* oxidase; lactate dehydrogenase, aldolase, glucose 6 phosphate dehydrogenase (and other glycolytic enzymes), as well as levels of serum GSH, and also to identify the balance of NO species in inflammation, resolved and unresolvable (GSNO, albumin/NO complexes). As mentioned earlier, some notable corroboratory pioneering studies of the impact of Cbl, or its deficiency, on the key inflammatory cytokines TNFα [1] and interleukin-6 [2,54], as well as on the growth factors EGF [1] and NGF [3] already exist. If, as the new paradigm and evidence suggests, such relationships are really an outcome of Cbl NO central regulation [7], these studies should be extended in the light of this hypothesis. It may be predicted that Cbl will also regulate most other growth factors, such as transforming growth factor β1, essential for the resolution of inflammation, basic fibroblast growth factor (bFGF) or vascular endothelial growth factor, which has pleiotropic Sp1, also the TCII transcription factor, in its promoter region [55]. This might have implications for the anti-angiogenic treatment of cancer with Cbl/interferon. Such a combination might additionally fully alert the immune system to the presence of tumour cells that normally evade surveillance, and thus promote endogenous existing means for tumour eradication via strong promotion of iNOS NO production. The posited central Cbl–NO regulation as outlined in The Return of the Scarlet Pimpernel may also provide an alternative explanation for the lack of toxicity and efficacy of exogenous NOCbl/interferon β demonstrated in cancer [56], a study that was critiqued in A Scarlet Pimpernel for the Resolution of Inflammation? [6] Exogenous NOCbl was used as a vehicle for the theoretical safe delivery of NO to completely eradicate ovarian tumours. NOCbl uptake by tumours was promoted by interferon β, which normally upregulates TCII receptors in inflammation [56], promoting increased intracellular arrival of H_2_OCbl. However, this study used no control OHCbl/interferon β and therefore cannot prove that the impressive results attributed to exogenous NO alone were not also effected by other Cbls endogenously [6]. Yet, there is both laboratory and clinical evidence for therapeutic effects of high-dose Cbls — CNCbl, OHCbl, MeCbl — in cancer [4,5,57–63]. Some in vitro evidence even demonstrates that the combination of Cbl and interferon β is synergistic in astrocyte gliosis [64]. However, no explanation of the fundamental mechanism of such anti-oncogenic Cbl effects has been published until now.

With renewed hindsight, the extraordinary curative high doses of NOCbl injected into mice with tumours in that study — equivalent to 12 g daily for nearly 3 weeks in humans — may have been rapidly converted to GSNO, and OHCbl, in the circulation, and thence to GSCbl intracellularly, and probably also promoted additional endogenous high NO production, as well as providing the native intracellular Cbl tools (GSCbl/AdoCbl/MeCbl) to deploy it without host toxicity, and may, in fact, be unwitting proof of the Scarlet Pimpernel hypotheses.
